# The Impact of Social Media Self-Presentation and Anxiety on Ruminative Exploration Among Women Across Diverse Racial and Ethnic Groups

**DOI:** 10.3390/healthcare14132016

**Published:** 2026-07-07

**Authors:** Julia Zavala, Terrence Calistro, Taylor Harrington, Rebecca C. Trenz

**Affiliations:** 1 Department of Psychology, Mercy University, Dobbs Ferry, NY 10522, USA; tcalistro@mercy.edu (T.C.); tharrington1@mercy.edu (T.H.); 2 Department of Psychology, Clarkson University, Potsdam, NY 13699, USA; rtrenz@clarkson.edu

**Keywords:** social media self-presentation, anxiety, ruminative exploration, emerging adults, diverse racial/ethnic groups

## Abstract

**Highlights:**

**What are the main findings?**

- Deep and authentic social media self-presentation (SMSP) predicts lower ruminative exploration for Black women, but not for Latina or White women.
- Anxiety predicts higher ruminative exploration for women, but lacks an interaction with SMSP.

**What are the implications of the main findings?**

- Deep and authentic SMSP is associated with less ruminative exploration for Black women.
- Anxiety increases ruminative exploration despite how women present themselves on social media.

**Abstract:**

**Background**: Ruminative exploration is a maladaptive form of identity development and is related to higher rates of anxiety and depression. Few researchers have examined how the four dimensions of social media self-presentation (breadth, depth, positivity, authenticity) impact ruminative exploration; much of the literature focuses on identity coherence or identity clarity. Furthermore, the social diversification hypothesis supports that social media is used differently across racial/ethnic groups. The current study aimed to understand how social media self-presentation predicted ruminative exploration while examining the role of anxiety among women (18–29 years old) from diverse backgrounds. **Method**: The study design was cross-sectional and used convenience sampling. Participants (*N* = 225) completed surveys including the Social Media Self-Presentation Scale (SMSPS), the State-Trait Inventory for Cognitive and Somatic Anxiety, and the Dimensions of Identity Development Scale. **Results**: Overall, breadth and authenticity of social media self-presentation predicted lower ruminative exploration, but anxiety predicted higher ruminative exploration. There was no interaction between anxiety and any of the four SMSPS dimensions. Distinct patterns emerged when examining social media self-presentation on ruminative exploration by racial/ethnic groups. Deep and authentic presentation predicted lower ruminative exploration for Black women. Posting positive content predicted higher ruminative exploration for White women. For Latina women, none of the SMSPS dimensions predicted ruminative exploration. For each racial/ethnic group, anxiety predicted higher ruminative exploration, but there was no interaction between any of the SMSPS dimensions and anxiety on ruminative exploration. **Conclusions:** Findings indicate that sharing deeply and authentically may support less ruminative exploration, particularly for Black women. However, anxiety’s impact on ruminative exploration is not moderated by social media self-presentation.

## 1. Introduction

Data has shown that 84% of emerging adults ages 18–29 years old use social media [1]. In the United States a larger percentage of women and Black and Hispanic individuals use some social media sites more than their male and White counterparts [2]. Users of social media often share their life experiences, where some posts may reflect one’s true experience while others may be exaggerated in an effort to be seen favorably. Those who are preoccupied with how they present themselves on social media tend to experience more anxiety and depression [3]. Specifically, the appearance-related social media consciousness, or continuous monitoring of how they appear and are perceived on social media may lead to depressive symptoms and maladaptive behaviors [4]. Gender differences in how social media impacts internalizing behaviors, such as depression and anxiety, have been mixed where some have found that females experience more internalizing behaviors than males after using social media [5], while others have found these experiences to be higher among males [6], and some have found no gender differences [3]. Both anxiety and social media self-presentation have been associated with identity development [7,8]. Specifically, sharing content on social media can shape one’s identity, but how self-presentation on social media is related to ruminative exploration, is not clear [7]. Ruminative exploration, or brooding, is a dimension of identity development which focuses on persistent attentiveness to oneself due to a perceived or actual threat to the self, questioning oneself, and having a difficult time making identity commitments [8]. The perception of having to live up to high standards or not feeling supported by others can lead one to ruminate, in turn, contributing to hopelessness and depression [9]. Using the Dimensions of Identity Development Scale (DIDS), researchers have found that ruminative exploration is higher among women [8] and has been related to higher levels of depression and anxiety symptoms and lower levels of self-esteem [8,10]. With the prevalence of social media use, researchers have attempted to examine the role of social media self-presentation on identity development, with very few focusing on ruminative exploration. The impact of social media self-presentation on ruminative exploration is of particular interest among emerging adults as this developmental timeframe is characterized as a period of change where individuals explore many different aspects of self, others, and their world [11]. Maladaptive identity formation during emerging adulthood may have lasting impacts that extend beyond this normative developmental period of change. Conversely, understanding how social media self-presentation may support adaptive identity formation, particularly buffering against ruminative exploration is important to understand, especially among women, as the literature demonstrates mixed findings for how women are impacted by social media self-presentation [3,5,6] and that they tend to ruminate more [8]. Furthermore, social media platforms create a space where users can edit their appearance and by doing so, researchers have found that this inauthentic self-presentation is linked to lower levels of self-perceived attractiveness and self-esteem and mediated by high levels of self-objectification and physical appearance comparisons [12]. Women tend to self-objectify more than men [13] and this has been linked to rumination [14]. Therefore, understanding the relationship between social media self-presentation and ruminative exploration among women is imperative.

Furthermore, the social diversification hypothesis proposes that non-White participants may use social media differently than their White counterparts as they tend to have less social capital offline and therefore may be motivated to go on social media to acquire social capital [15]. Given this theory, the relationship between social media self-presentation and ruminative exploration may depict unique patterns among social media users of different races and ethnicities especially since Asian, Black, and Latino Americans in the US create new content on social media more than their White counterparts [2,16].

Self-presentation is characterized as an individual’s sharing of information about themselves to others and is motivated by obtaining favorability and achieving some level of self-fulfillment [17]. Consistent with this theory, social media self-presentation is motivated by these two desires, but social media platforms have changed the way in which the user self-presents [18], as a person can manipulate their own profile to gain favor from their followers [19,20,21,22]. Due to the amount of engagement on social media by emerging adults, particularly women, social media self-presentation has become a salient component of one’s identity development during this period [7]. The four-dimensional model of social media self-presentation consists of breadth, depth, positivity, and authenticity [23]. Breadth refers to how much information a person presents online, depth refers to how intimate a person is with the shared information, positivity indicates the favorability of the information presented, and authenticity denotes the accuracy of information.

Despite the extent of social media use among emerging adults and the potential for it shaping identity development among this group, there is a dearth of research on the impact of social media self-presentation on ruminative exploration. To the authors’ knowledge, only Kawamoto has studied social media self-presentation and ruminative exploration; however, only the depth dimension of self-presentation among a group of Japanese emerging adults was investigated [7]. Their results showed that surface-level self-presentation (i.e., sharing of daily activities and life events) resulted in a positive correlation with ruminative exploration, but deeper self-presentation did not correlate with ruminative exploration, suggesting that shallow self-presentation can be problematic. In contrast, Yang and colleagues found that a racially diverse group of emerging adults who presented themselves in depth on social media had low identity clarity, but these researchers did not assess ruminative exploration [23]. In both of these studies, there were no differences between male and female participants.

Furthermore, researchers have found that those presenting themselves positively resulted in higher identity clarity for mindful individuals, but less mindful individuals had lower identity clarity [23]. The latter could lead to rumination where social media users second guess what they post, preventing identity clarity [23]. In regard to the breadth and authenticity dimensions of social media self-presentation, these two constructs were not related to identity clarity [23]. However, among a group of emerging adults who mostly identified as Latino Americans (72.7%), researchers found that identity coherence is related to authentic (i.e., accurate representation of self) self-presentation [24]. Identity coherence is a characteristic of lower ruminative exploration [25]. On the other hand, those categorized as displaying identity confusion, a characteristic of ruminative exploration [25], typically presented themselves inauthentically on social media [24]. These results were not different between male and female participants. Others have found that lower authentic online self-presentation resulted in rumination among high school seniors in China, but did not focus on ruminative exploration or consider gender differences [26]. To date, few studies have examined the relationship between social media self-presentation and ruminative exploration, specifically. Among those who have, the results remain unclear as they focused on only one dimension of social media self-presentation [7]. Furthermore, the existing literature does not consistently incorporate gender or race/ethnicity into their models, further delineating a gap in our understanding of the relationship between the dimensions of social media self-presentation and ruminative exploration across genders and race/ethnicities. Given the prevalence of social media use among emerging adult women, the current study sought to further explore the relationship between social media self-presentation and ruminative exploration among a racially/ethnically diverse group of women who are emerging adults in college.

The aim of the current study was to examine the relationship between social media self-presentation (breadth, depth, positivity, and authenticity) and ruminative exploration. An additional goal was to understand how trait anxiety intersects with the four dimensions of social media self-presentation on ruminative exploration. These factors are of particular interest as they may be predictive of ruminative exploration which could lead to negative outcomes, such as depression. Given the literature on social media self-presentation, anxiety, and ruminative exploration among racially/ethnically diverse women, the following exploratory research questions will be investigated:

R1: Do the four dimensions of social media self-presentation, trait anxiety, and demographic variables correlate with ruminative exploration among a racially/ethnically diverse group of women who are emerging adults?

R2: Do the four dimensions of social media self-presentation predict ruminative exploration among a racially/ethnically diverse group of women who are emerging adults?

R3: Does anxiety predict ruminative exploration among a racially/ethnically diverse group of women who are emerging adults?

R4: Do the four dimensions of social media self-presentation intersect with trait anxiety to predict ruminative exploration among a racially/ethnically diverse group of women who are emerging adults?

## 2. Materials and Methods

### 2.1. Participants

Study participants were recruited from a Hispanic Serving Institution in the Northeast region of the United States. Inclusion criteria were enrollment at the University and aged 18–29 years old. The final sample consisted of 225 female emerging adults with a mean age of 20.82 (*SD* = 2.92). Participants identified as Latino/Latina (39.6%), White (29.3%), Black (25.8%), Asian (7.1%), or other (5.3%). See Table 1 for a full summary of demographic variables.

### 2.2. Study Design and Procedure

The study design was cross-sectional. Data collection took place from May 2018 through September 2019. Participants were recruited using convenience sampling. One hundred surveys were collected in-person where 21 responses were collected via paper surveys and 79 were collected via tablets that linked to the survey online through REDCap (https://project-redcap.org/). These first 100 participants received 5 USD for their participation. Subsequent responses were collected remotely via the online survey link to RedCap and these participants were not remunerated. Participants first provided consent then completed a demographic questionnaire, a social media use questionnaire, Dimensions of Identity Development Scale, Social Media Self-Presentation Scale, and State-Trait Inventory for Cognitive and Somatic Anxiety Scale. It took approximately 15 min for the participants to complete the study. This research was approved by the University’s Institutional Review Board.

### 2.3. Measures

#### 2.3.1. Demographics

The demographic questionnaire consisted of six questions that asked the participants to indicate their sex (male or female), year in school (freshman, sophomore, junior, or senior), race/ethnicity (Black, Latina, White, Asian, or other), sexual orientation (heterosexual, homosexual, bisexual, or other), residential status (on campus in the dorm, on campus in the hotel, with parents and/or family, or independent of parents and/or family), and socio-economic status (lower class, middle class, upper-middle class, or upper class).

#### 2.3.2. Social Media Use

The Social Media Use scale measured social media use in both minutes per day and times checked per day on each of the following platforms: Instagram, Snapchat, YouTube, Facebook, Twitter, LinkedIn, Tumbler, and “other” category.

#### 2.3.3. Ruminative Exploration

The 25-item Dimensions of Identity Development Scale (DIDS) was used [8]. This scale consists of five dimensions of identity: (1) commitment making, (2) identification with commitment, (3) exploration in breadth, (4) exploration in depth, and (5) ruminative exploration. For the purpose of the current study, only the ruminative exploration dimension was used as the dependent variable. It consisted of five items where participants responded to each question on a five-point Likert scale from one (strongly disagree) to five (strongly agree). The instrument was scored by summing the item responses for ruminative exploration. Higher scores indicate higher levels of ruminative exploration. This scale has been found to have high internal construct validity and high reliability, with all Cronbach’s alphas for each dimension at 0.80 or higher [8]. The high reliability (i.e., based on Cronbach’s alphas) was also found within this study for all the dimensions aside from exploration in depth: commitment making (0.91), identification with commitment (0.88), exploration in breadth (0.87), exploration in depth (0.58), and ruminative exploration (0.87). The Cronbach’s alpha that was of particular interest for this study was for ruminative exploration as it was the dimension that was included as the dependent variable in the analyses below.

#### 2.3.4. Self-Presentation on Social Media

The Social Media Self-Presentation Scale (SMSPS) was used [23,27]. The four dimensions included in the scale were: (1) breadth, four items; (2) depth, five items; (3) positivity, three items; and (4) authenticity, five items. Participants responded to each item on a five-point Likert scale from one (strongly disagree) to five (strongly agree). All three items for the positivity dimension and one item for authenticity were reverse scored. Items for each dimension were totaled for individual dimension scores. Internal consistency for each dimension has been found to be above 0.70 [27]. The high reliability (i.e., based on Cronbach’s alphas) was also found within this study for all the dimensions aside from authenticity: breadth (0.78), depth (0.79), positivity (0.83), and authenticity (0.60).

#### 2.3.5. Anxiety

The State-Trait Inventory for Cognitive and Somatic Anxiety Scale (STICSA) was used to measure participant trait anxiety [28]. This scale has 21 items that ask participants to rate their cognitive and somatic symptoms of anxiety on a four-point Likert scale from one (not at all) to four (very much so). Split-half reliability for cognitive and somatic items on the trait scale was 0.87 and 0.84, respectively [28]. The STICSA-trait scale also has high convergent validity with the STAI-trait scale [29]. The internal consistency for the instrument in this study was high, with a Cronbach alpha of 0.92.

## 3. Results

The descriptive statistics for the continuous variables: social media self-presentation breadth, depth, positivity, and authenticity; trait anxiety; and ruminative exploration are depicted in Table 2.

Frequency of daily social media use showed that the average number of times per day that participants checked Instagram, Snapchat, Facebook, Twitter, and YouTube was 37.84 (*SD* = 30.81). The average number of minutes spent on those sites per day was 196.22 (*SD* = 235.05).

Table 3 shows the Pearson coefficients that examine the bivariate associations between demographic variables, SMSPS dimensions, social media usage measured in average number of minutes spent on social media and number of times checking social media platforms per day, trait anxiety levels, and ruminative exploration. Categorical demographic variables such as race/ethnicity were dummy-coded so the variables could be included in the correlation analysis. The correlations that include the dummy coded demographic variables were analyzed using point-biserial coefficients. Point-biserial correlations examine the associations between a numeric/continuous variable and a binary/dummy coded variable [30]. Notable associations were that SMSPS: positivity (*r* = 0.22, *p* < 0.01) and trait anxiety (*r* = 0.43, *p* < 0.01) were significantly positively correlated with ruminative exploration.

Four regression analyses were conducted to determine if each social media self-presentation dimension, anxiety, and the interaction between these two variables significantly predicted ruminative exploration among women. In these four regression models, the predictors were mean-centered in order to reduce multicollinearity that can occur within moderation analysis. There was evidence of absence of multicollinearity since the VIF values of all the predictors within the models were less than five [31]. When all female participants were combined, regardless of race/ethnicity, all four regression models were statistically significant (all *ps* < 0.001), explaining between 19.5% and 21.3% of the variance in ruminative exploration. In Model 1, SMSPS Breadth was a significant negative predictor of ruminative exploration, *B* = −0.214, *SE* = 0.087, β = −0.154, *t* = −2.457, *p* = 0.015, indicating that greater breadth of self-presentation was associated with lower levels of ruminative exploration. Trait anxiety was a strong positive predictor, *B* = 0.173, *SE* = 0.025, β = 0.444, *t* = 7.050, *p* < 0.001. The Breadth × Anxiety interaction was not significant, *B* = −0.006, *SE* = 0.008, β = −0.052, *t* = −0.830, *p* = 0.407. In Model 2, SMSPS Depth was not a significant predictor, *B* = −0.112, *SE* = 0.082, β = −0.088, *t* = −1.362, *p* = 0.175, whereas anxiety again significantly predicted ruminative exploration, *B* = 0.175, *SE* = 0.025, β = 0.448, *t* = 6.968, *p* < 0.001. The Depth × Anxiety interaction was not significant, *B* = −0.001, *SE* = 0.007, β = −0.009, *t* = −0.134, *p* = 0.894. In Model 3, SMSPS Positivity was marginally significant, *B* = 0.253, *SE* = 0.135, β = 0.122, *t* = 1.876, *p* = 0.062, while anxiety remained a significant positive predictor, *B* = 0.154, *SE* = 0.025, β = 0.393, *t* = 6.080, *p* < 0.001. The Positivity × Anxiety interaction was not significant, *B* = 0.012, *SE* = 0.010, β = 0.077, *t* = 1.196, *p* = 0.233. Finally, in Model 4, SMSPS Authenticity significantly and negatively predicted ruminative exploration, *B* = −0.238, *SE* = 0.097, β = −0.155, *t* = −2.466, *p* = 0.014, and anxiety again emerged as a robust positive predictor, *B* = 0.177, *SE* = 0.025, β = 0.452, *t* = 7.132, *p* < 0.001. The Authenticity × Anxiety interaction was not significant, *B* = 0.003, *SE* = 0.007, β = 0.025, *t* = 0.390, *p* = 0.697. See Table 4 for regression statistics for Models 1–4.

When analyses were conducted separately by racial/ethnic group, distinct patterns emerged. Among Latina participants (*n* = 85), anxiety was an extremely strong positive predictor across Models 5–8 in Table 4 (*Bs* ≈ 2.2, all *ps* < 0.001), whereas none of the social media self-presentation dimensions—breadth, depth, positivity, or authenticity—significantly predicted ruminative exploration (all *ps* > 0.07). Among Black participants (*n* = 51), several social media self-presentation dimensions were significant or marginally significant predictors of ruminative exploration. Breadth was marginally significant, *B* = −0.337, *p* = 0.051, Depth was a significant negative predictor, *B* = −0.303, *p* = 0.046, and Authenticity was also a significant negative predictor, *B* = −0.343, *p* = 0.036. In these models, anxiety remained a significant positive predictor (e.g., in the Depth model, *B* = 0.160, *p* = 0.006). See Models 9–12 in Table 4. For White participants (*n* = 61), anxiety was again a significant positive predictor (e.g., *B* = 0.189, *p* < 0.001 in Model 13). SMSPS Positivity was marginally significant, *B* = 0.476, *p* = 0.050, suggesting that higher positivity in self-presentation was associated with higher ruminative exploration in this group. Breadth, Depth, and Authenticity were not significant predictors. See Models 13–16 in Table 4. These analyses were not conducted for women who identified as Asian (*n* = 16, 7.1%) or Other (*n* = 12, 5.3%) as there were too few participants in those groups. In all the ethnicity subgroup regression models, the predictors were also mean-centered in order to reduce multicollinearity that can occur within moderation analysis. There was evidence of absence of multicollinearity since the VIF values of all the predictors within the models were less than five [31].

Because the four dimensions of social media self-presentation are theoretically related but conceptually distinct, each dimension was first modeled separately to examine its dimension-specific association with the outcome. This approach was selected to enhance interpretability and to avoid obscuring potentially meaningful associations by partialling out shared variance among related facets of the same broader construct. A simultaneous model including all four self-presentation dimensions was also examined as a sensitivity analysis to evaluate whether any associations remained after accounting for the overlap among the dimensions. An a priori power analysis conducted in G*Power 3.1 indicated that, for a linear multiple regression model with three predictors (i.e., the moderation models), a medium effect size (f^2^ = 0.15), α = 0.05, and power = 0.80, a minimum sample size of 77 participants was required. The achieved power for this model was 0.802. The overall sample size (*N* = 255) and the Hispanic subgroup (*n* = 89) exceeds the minimal sample, but not the Black and White subgroups. Given the number of regression models estimated, the analyses should be interpreted as exploratory and hypothesis-generating. We therefore focus on the pattern, direction, and magnitude of associations rather than treating any single *p*-value as definitive evidence. Marginal findings are interpreted cautiously and are not used as the sole basis for substantive conclusions.

Regression diagnostics in Table 5 were examined across all 16 models to evaluate independence of errors, residual normality, homoscedasticity, influential residuals, and multicollinearity. Durbin–Watson values ranged from 1.70 to 2.38, suggesting no substantial evidence of autocorrelation in the residuals. Standardized residuals ranged from −2.89 to 2.23 across models, with all values falling within the conventional ±3.00 threshold, indicating no extreme residual outliers. Visual inspection of the standardized residual histograms and normal P-P plots indicated that the residuals were approximately normally distributed across models, with only minor deviations from the diagonal line in some subgroup models. Scatterplots of standardized residuals against standardized predicted values did not show clear funneling, curvature, or systematic patterns, supporting the assumption of homoscedasticity. Multicollinearity diagnostics were also acceptable, with tolerance values ranging from 0.657 to 0.984 and variance inflation factor values ranging from 1.02 to 1.52. Condition indices ranged from 1.16 to 2.51, providing no evidence of problematic multicollinearity. Overall, the regression diagnostics suggested that the models adequately met the assumptions for linear regression.

## 4. Discussion

The current study examined if social media self-presentation and anxiety predict ruminative exploration. It further explored the intersection of social media self-presentation and anxiety on ruminative exploration. These analyses were conducted among a group of racially/ethnically diverse women who were emerging adults in college and then analyzed separately to understand if these relationships differed among different racial/ethnic identities. Across all models and groups, anxiety consistently emerged as the most robust predictor of ruminative exploration, highlighting a strong association between trait anxiety and ruminative exploration which is consistent with research that has used the Dimensions of Identity Development Scale (DIDS) to measure ruminative exploration [8,10]. In the overall sample that included all women regardless of race/ethnicity, greater breadth and authenticity in social media self-presentation appeared to be associated with lower ruminative exploration. This finding aligns with research conducted by Michikyan [24] who found that authentic social media self-presentation was related to higher identity coherence, a construct closely associated with lower rumination [25]. Our results that showed that breadth resulted in lower ruminative exploration is a novel finding, as work conducted by Yang et al. did not find an association between breadth and identity clarity [23]. These contrasting results may be due to the fact that identity clarity and ruminative exploration are two distinct constructs. Furthermore, we included a sample of only women who were emerging adults to better understand these relationships among this demographic, whereas they controlled for gender in their regression.

Moreover, the findings in the present study varied by racial/ethnic groups. For Black women, deeper and more authentic self-presentation was associated with significantly lower ruminative exploration indicating that presenting oneself on social media in a deep and authentic way may predict less feelings of brooding. Researchers have found that Black individuals who have a strong racial identity are more resilient and experience lower rates of anxiety and depression [32]. We did not collect data on racial identity and it may be that racial identity might contribute to feeling comfortable enough to share deeply and authentically on social media, an area of research that should be explored further. For White women, higher positivity in self-presentation showed a marginal association with increased ruminative exploration. This finding aligns with research that found that less mindful students who present themselves positively on social media tend to have lower identity clarity which could potentially lead to rumination as explained by Yang et al. [23]. The theoretical implication of this finding also aligns with the fragmentation hypothesis, which proposes that the varying identities one portrays online fragments their personalities making it difficult to develop identity clarity [33]. For instance, when presenting themselves positively, they may be presenting an alternate variation of their self, impeding their identity development and thus ruminating on the positive presentation they present. Furthermore, these findings may be attributed to the objectification theory which states that self-objectification or constant self-surveillance can lead to higher rates of body shaming [34], which is a construct that has been linked to rumination [14]. Black women experience lower levels of self-objectification than their White counterparts [34]. This may contribute to the explanation for why the Black women in the current sample who presented themselves more deeply and authentically experienced lower levels of ruminative exploration in contrast to the White women who presented themselves more positively and had higher levels of ruminative exploration. Future research should account for self-objectification to better understand its role in the aforementioned relationships. Furthermore, self-objectification has been linked with different platforms and not others, i.e., with Instagram but not Facebook [35]. Future research should explore how women self-present on various platforms to better understand the impact it may have on ruminative exploration.

The current study found that for Latina women, social media self-presentation dimensions did not significantly predict ruminative exploration beyond the strong influence of anxiety. Notably, no significant interaction effects were observed between social media self-presentation dimensions and anxiety in any model, indicating that the associations between the dimensions of social media self-presentation and ruminative exploration were independent of anxiety levels. However, researchers have found appearance anxiety to be related to social media use [3]. This study measured trait anxiety; future research should explore the moderating role of appearance anxiety on social media self-presentation and ruminative exploration. Despite this, overall, the findings suggest that although trait anxiety is the primary driver of ruminative exploration, certain dimensions of social media self-presentation, particularly authenticity and depth, may be predictive of lower levels of ruminative exploration for some groups, specifically Black women in this sample and positive self-presentation may be predicative of higher levels of ruminative exploration among White women in this sample.

To the authors’ knowledge, the present study is novel in examining social media self-presentation and trait anxiety as predictors of ruminative exploration among racially/ethnically diverse women who are emerging adults in college. A noteworthy strength of the current study is the diversity of study participants, where almost three-quarters identified as non-White allowing to generalize these results to more diverse populations. In addition, the majority of study participants were non-traditional students in that two-thirds were commuter students. Given these demographic factors, the current study findings are a unique contribution to the social media use and ruminative exploration literature among emerging adults.

Despite these important study findings, there are several limitations. This study is cross-sectional and does not capture how social media self-presentation is related to ruminative exploration over time and limits the ability to interpret any causal relationships among our variables. Therefore, future research should test these relationships using longitudinal designs. Another limitation is that participants were recruited from a single Hispanic Serving Institution which may impact the generalizability to other populations of emerging adults who are women. Additionally, the sample included only women; future research should test these relationships among men and nonbinary participants. Other factors such as racial identity or online discrimination were not examined and could potentially contribute to these outcomes in unique ways. Lastly, the study data presented herein was collected pre-COVID in mid-2018 to late 2019 and TikTok had not yet gained popularity in the United States. Despite the changes in social media use since then, TikTok is only the fourth most used platform, with YouTube leading, followed by Facebook, and Instagram [2] and the use of all three of these were recorded in the present data. Therefore, this study has value in that it is informative of a pre-COVID marker of this phenomenon and may serve as the basis for exploring these predictors in contemporary contexts that are illustrative of current social media use trends over time. Despite these limitations, this research provides a nuanced picture of the relationship between social media self-presentation and ruminative exploration among a group of racially and ethnically diverse women who are emerging adults. The findings indicate that social media self-presentation can be a predictive factor of lower ruminative exploration and could help to inform interventions and recommendations for healthy social media use among women. This study is limited to one sample; therefore, future research should test interventions that may support healthy social media use across diverse regions and populations in the US. In addition, there is an increased risk of Type I error due to the number of regression models estimated. Although the models were theoretically motivated, the analyses were exploratory and were not designed to provide confirmatory evidence for each individual association. Marginal findings, in particular, should be interpreted cautiously. Future research should replicate these patterns using larger samples, preregistered hypotheses, and analytic strategies that formally address multiplicity.

## 5. Conclusions

In this study, among the sample of emerging adult women, overall, those who presented themselves with more breadth, depth, and authenticity on social media had lower levels of ruminative exploration. These associations differed among different groups of race and ethnicity. Black women who shared more deeply and authentically on social media had lower rates of ruminative exploration, but this was not evident among Latina or White women. Conversely, White women who scored higher on positive self-presentation had higher levels of ruminative exploration which was not true for Black or Latina women. These associations may be explained by differences in how women of different races and ethnicities self-objectify online, use social media for social capital, or in the strength of their own identity. Future research should further explore how these theoretical constructs contribute to the link between social media self-presentation and ruminative exploration.

## Figures and Tables

**Table 1:** Demographic variables summary statistics (*N* = 225).

Variables		*M*	*SD*
Age		20.82	2.92
Variables	Categories	*n*	*%*
Academic year	Freshman	81	37.5%
	Sophomore	33	15.3%
	Junior	44	20.4%
	Senior	58	26.9%
Race/Ethnicity	Black	58	25.8%
	Latina	89	39.6%
	White	66	29.3%
	Asian	16	7.1%
	Other	12	5.3%
Sexual Orientation	Heterosexual	180	80.4%
	Homosexual	5	2.2%
	Bisexual	26	11.6%
	Other	13	5.8%
Residential Status	On campus (Dorm)	31	14.0%
	With parents and/or family	158	71.2%
	Independent of parents and/or family	33	14.9%
SES	Lower class	53	23.7%
	Middle class	148	66.1%
	Upper-Middle class	22	9.8%
	Upper class	1	0.4%

**Table 2:** Mean and standard deviations for predictor and outcome variables.

Variables	*M*	*SD*
SMSPS: Breadth	12.50	3.48
SMSPS: Depth	10.52	3.76
SMSPS: Positivity	5.33	2.32
SMSPS: Authenticity	16.34	3.14
Trait Anxiety	40.79	12.36
Ruminative Exploration	14.64	4.86

**Table 3:** Correlation matrix of main study variables.

Variable	1	2	3	4	5	6	7	8	9	10	11	12	13
1. Age	—												
2. Black	−0.09	—											
3. Latina	0.06	−0.35 **	—										
4. White	0.09	−0.29 **	−0.46 **	—									
5. Asian	−0.07	−0.12	−0.19 **	−0.10	—								
6. SMSPS: Breadth	0.07	−0.12	−0.23 **	0.45 **	−0.07	—							
7. SMSPS: Depth	0.08	0.15 *	−0.18 *	0.13	0.01	0.39 **	—						
8. SMSPS: Positivity	0.01	0.10	−0.10	0.09	0.04	0.21 **	0.58 **	—					
9. SMSPS: Authenticity	0.06	−0.12	−0.04	0.14 *	0.09	0.42 **	0.31 **	0.09	—				
10. Trait Anxiety	−0.02	−0.16 *	0.07	0.19 **	−0.04	0.10	0.17 *	0.22 **	0.10	—			
11. SMU Times	0.02	0.02	−0.02	0.01	−0.04	0.17 *	0.19 *	0.07	−0.11	0.06	—		
12. SMU Minutes	0.02	0.12	−0.06	−0.03	−0.03	0.13	0.06	0.01	0.07	0.03	0.41 **	—	
13. Ruminative Exploration	−0.11	−0.05	0.02	0.06	0.07	−0.11	−0.01	0.22 **	−0.11	0.43 **	0.06	0.08	—

Note. Race variables were dummy coded (1 = yes, 0 = no). SMSPS = Social Media Self-Presentation Scale. SMU = Social Media Use. * *p* < 0.05. ** *p* < 0.01.

**Table 4:** Regression table of individual SMSPS dimensions, trait anxiety, and their interactions predicting ruminative exploration.

Variable	Full Sample	Latina	Black	White
	Model 1	Model 5	Model 9	Model 13
Intercept	14.65 *** [14.05, 15.25]	14.34 *** [13.33, 15.34]	14.62 *** [13.32, 15.91]	15.04 *** [13.65, 16.42]
SMSPS: Breadth	−0.21 * [−0.39, −0.04]	−0.08 [−0.43, 0.26]	−0.34 [−0.68, 0.00]	−0.21 [−0.58, 0.16]
Trait Anxiety	0.17 *** [0.12, 0.22]	0.22 *** [0.14, 0.31]	0.13 * [0.01, 0.25]	0.19 *** [0.09, 0.29]
SMSPS: Breadth × Trait Anxiety	−0.01 [−0.02, 0.01]	0 [−0.03, 0.03]	−0.01 [−0.04, 0.02]	−0.01 [−0.05, 0.02]
R^2^	0.21	0.31	0.19	0.24
Adj R^2^	0.2	0.29	0.14	0.21
F	18.261 ***	12.338 ***	3.685 *	6.049 ***
	Model 2	Model 6	Model 10	Model 14
Intercept	14.62 *** [14.02, 15.23]	14.38 *** [13.40, 15.36]	15.21 *** [13.89, 16.54]	14.42 *** [13.28, 15.56]
SMSPS: Depth	−0.11 [−0.27, 0.05]	−0.09 [−0.38, 0.19]	−0.30 * [−0.60, −0.01]	0.05 [−0.27, 0.37]
Trait Anxiety	0.17 *** [0.13, 0.22]	0.23 *** [0.15, 0.31]	0.16 ** [0.05, 0.27]	0.15 *** [0.07, 0.24]
SMSPS: Depth × Trait Anxiety	0 [−0.01, 0.01]	−0.01 [−0.03, 0.02]	−0.02 [−0.04, 0.01]	0 [−0.02, 0.03]
R^2^	0.2	0.32	0.2	0.21
Adj R^2^	0.18	0.29	0.14	0.17
F	16.287 ***	12.540 ***	3.788 *	5.134 **
	Model 3	Model 7	Model 11	Model 15
Intercept	14.54 *** [13.93, 15.15]	14.56 *** [13.59, 15.53]	14.75 *** [13.37, 16.13]	14.37 *** [13.30, 15.44]
SMSPS: Positivity	0.25 [−0.01, 0.52]	0.35 [−0.11, 0.81]	0.16 [−0.43, 0.74]	0.48 * [0.00, 0.95]
Trait Anxiety	0.15 *** [0.10, 0.20]	0.22 *** [0.15, 0.30]	0.14 * [0.02, 0.25]	0.13 ** [0.05, 0.21]
SMSPS: Positivity × Trait Anxiety	0.01 [−0.01, 0.03]	−0.01 [−0.04, 0.03]	0 [−0.05, 0.05]	0.01 [−0.03, 0.04]
R^2^	0.21	0.33	0.12	0.28
Adj R^2^	0.2	0.31	0.07	0.24
F	17.891 ***	13.280 ***	2.163	7.364 ***
	Model 4	Model 8	Model 12	Model 16
Intercept	14.61 *** [14.01, 15.20]	14.38 *** [13.45, 15.32]	14.67 *** [13.41, 15.94]	14.27 *** [13.18, 15.36]
SMSPS: Authenticity	−0.24 * [−0.43, −0.05]	−0.27 [−0.57, 0.03]	−0.34 * [−0.66, −0.02]	0.24 [−0.23, 0.71]
Trait Anxiety	0.18 *** [0.13, 0.23]	0.22 *** [0.14, 0.30]	0.13 * [0.02, 0.24]	0.15 *** [0.07, 0.23]
SMSPS: Authenticity × Trait Anxiety	0 [−0.01, 0.02]	−0.01 [−0.03, 0.01]	−0.01 [−0.04, 0.01]	0.02 [−0.01, 0.05]
R^2^	0.21	0.34	0.22	0.26
Adj R^2^	0.2	0.32	0.17	0.23
F	18.156 ***	13.999 ***	4.373 **	6.816 ***

Note. 95% confidence intervals are in brackets. * *p* < 0.05, ** *p* < 0.01, *** *p* < 0.001.

**Table 5:** Regression diagnostic table for all 16 models.

Model	Sample	Model Tested	N	DW	Std. Residual Range	Max VIF/CI	Plot Diagnostics
1	Full sample	Breadth × STICSA	206	2.00	−2.62 to 1.96	1.02/1.16	Acceptable
2	Full sample	Depth × STICSA	206	1.98	−2.38 to 2.15	1.04/1.26	Acceptable
3	Full sample	Positivity × STICSA	206	1.96	−2.56 to 2.00	1.08/1.39	Acceptable
4	Full sample	Authenticity × STICSA	206	1.97	−2.73 to 2.20	1.03/1.22	Acceptable
5	Black	Breadth × STICSA	51	2.21	−2.27 to 1.80	1.25/1.83	Acceptable
6	Black	Depth × STICSA	51	2.17	−2.08 to 2.05	1.16/1.70	Minor P-P dev.; acceptable
7	Black	Positivity × STICSA	51	2.14	−2.11 to 1.84	1.11/1.61	Acceptable
8	Black	Authenticity × STICSA	51	2.38	−2.32 to 2.23	1.07/1.54	Acceptable
9	Latina	Breadth × STICSA	85	1.81	−2.76 to 1.95	1.31/1.80	Minor spread variation; acceptable
10	Latina	Depth × STICSA	85	1.87	−2.59 to 1.83	1.04/1.35	Minor spread variation; acceptable
11	Latina	Positivity × STICSA	85	1.86	−2.83 to 1.89	1.12/1.48	Minor spread variation; acceptable
12	Latina	Authenticity × STICSA	85	1.80	−2.89 to 1.96	1.11/1.40	Acceptable
13	White	Breadth × STICSA	61	1.85	−2.15 to 1.85	1.52/2.51	Acceptable
14	White	Depth × STICSA	61	1.80	−2.19 to 1.86	1.28/1.97	Acceptable
15	White	Positivity × STICSA	61	1.70	−1.98 to 2.21	1.26/1.86	Acceptable
16	White	Authenticity × STICSA	61	1.79	−1.98 to 1.85	1.23/1.80	Acceptable

Note. DW = Durbin–Watson; VIF = variance inflation factor; CI = condition index. Plot diagnostics summarize visual inspection of standardized residual histograms, normal P-P plots, and standardized residuals plotted against standardized predicted values. Minor deviations were retained as acceptable when no extreme standardized residuals, clear funneling, curvature, or systematic residual pattern was observed. Across models, tolerance values ranged from 0.657 to 0.984, VIF values ranged from 1.02 to 1.52, condition indices ranged from 1.16 to 2.51, and standardized residuals remained within ±3.00.

## Data Availability

The raw data supporting the conclusions of this article will be made available by the authors on request. The data are not publicly available due to privacy and ethical restrictions.
